# Gaze-based detection of mind wandering during audio-guided panorama viewing

**DOI:** 10.1038/s41598-024-79172-x

**Published:** 2024-11-14

**Authors:** Tiffany C. K. Kwok, Peter Kiefer, Victor R. Schinazi, Christoph Hoelscher, Martin Raubal

**Affiliations:** 1 Institute of Cartography and Geoinformation, ETH Zürich, Zurich, Switzerland; 2 Department of Psychology, Bond University, Gold Coast, Australia; 3 Chair of Cognitive Science, ETH Zürich, Zurich, Switzerland; 4 Lufthansa Systems FlightNav, Opfikon, Switzerland

**Keywords:** Computational science, Computer science

## Abstract

Unlike classic audio guides, intelligent audio guides can detect users’ level of attention and help them regain focus. In this paper, we investigate the detection of mind wandering (MW) from eye movements in a use case with a long focus distance. We present a novel MW annotation method for combined audio-visual stimuli and collect annotated MW data for the use case of audio-guided city panorama viewing. In two studies, MW classifiers are trained and validated, which are able to successfully detect MW in a 1-s time window. In study 1 (n = 27), MW classifiers from gaze features with and without eye vergence are trained (area under the curve of at least 0.80). We then re-validate the classifier with unseen data (study 2, n = 31) that are annotated using a memory task and find a positive correlation (repeated measure correlation = 0.49, *p* < 0.001) between incorrect quiz answering and the percentage of time users spent mind wandering. Overall, this paper contributes significant new knowledge on the detection of MW from gaze for use cases with audio-visual stimuli.

## Introduction

Audio guides can be effective in communicating information to users viewing a novel stimulus (e.g., tourists exploring a city panorama). When properly implemented, audio guides are capable of mimicking the experience of listening to a real person by playing back curated audio content. However, different to human guides, classic audio guides do not take into account the reaction of listeners (e.g., subtle changes in viewing behavior) and consequently are unable to detect a decrease in their interest with regard to the stimulus. Therefore, an audio guide that is unaware of a change in users’ interest could potentially disengage the user from using it. Although recreational activities, such as panorama viewing, are different from formal learning settings, it can be assumed that the user of an audio guide is motivated to learn about its content. Audio-visual interaction is fundamental to everyday tasks, particularly in naturalistic and educational settings, where visual stimuli often guide attention while audio content enhances understanding^1^. In this context, enhancing the responsiveness of audio guides to user behavior can generalize to broader educational tasks by supporting more dynamic and engaging learning experiences, similar to those in a classroom or interactive learning environment. Here, catching and maintaining the interest and attention of the user is essential for encouraging him/her to learn more about the stimulus. Thus, there is a need to have a system that takes proactive measures to regain users’ attention and bring them back to the main activity. Such a system could maintain users’ interest in continuing to learn more about the stimulus by inciting them to continue learning more about the stimulus. For example, changing the style of how the audio contents are presented or asking whether the users would like to switch to other topics that they might be more interested in.

Previous work suggests different ways of increasing the user experience (UX) of audio guides, including audio design^2^, content personalization^3^, story telling^4^, user self-localization^5^, sonic interaction design^6^ or locative audio^7^. However, even a very well-designed audio guide may not be able to keep users focused at all times because of situational factors and individual differences that are hard to foresee. When a user’s attention shifts from the audio guide to other unrelated thoughts, he/she will start mind wandering (MW), which has been defined as a cognitive state connected with the user’s disengagement of attention from a primary task^8^.

In this work, we focus on detecting MW with the use of eye-tracking technology. Eye-tracking can provide fast and natural ways to interact with different stimuli^9^ and has been successfully applied to explore a variety of cognitive states (including, e.g., attention detection^10^, intent prediction^11^, confusion detection^12^), and MW detection^13,14^. MW has also been detected from eye movements^13,14^. However, in contrast to numerous outdoor scenarios, previous work mainly focused on scenarios where users interact with objects (e.g., video displayed on a computer screen) located relatively close to the users. Thus, the suitability of gaze features, particularly features associated with eye vergence, for classifying MW remains unclear. With the goal of enabling an MW-aware audio guide, we present and evaluate MW classifiers that can be used as the back-end system of audio guide systems that are capable of interpreting users’ eye movements to recognize when they start MW in real time.

To achieve this goal, we first position our research within related work (“Related work” section), and then carry out the following steps and approach the research questions associated with them:

1. Detecting MW from eye movements (pre-study, n = 18, see “Pre-study: parameters for data annotation” section; and study 1, n = 27, see “Study 1: detecting mind wandering” section). Enabling the system to detect MW in real time is essential for the MW-aware audio guide. We replicated and adapted the data collection procedure and the state-of-the-art real time MW detection method proposed by Huang et al.^14^ for our scenario (i.e., for an audio-visual stimulus). The difference between the scenario used by Huang et al.^14^ and this work (see “Gaze-based detection of mind wandering/attention shift” section) leads to the following research question, which we address in this step: *(RQ1) Can eye vergence features be used for classifying MW in scenarios with longer focus distances?*
2. Re-validating the MW classifier (study 2, n = 31, see “Study 2: re-validation of the MW classifier in CAVE” section). Due to the design of the data annotation method of study 1, the training data did not contain any self-reported label as we did not want to artificially interrupt participants during data collection. In step 2, we, therefore, validate our MW classifier in a more realistic task (i.e., without interrupting the UX), by correlating answers to questions from an audio-related quiz with the percentage of time classified as MW. Study 2 addresses the research question: *(RQ2) Can MW be detected reliably for uninterrupted audio-guided panorama viewing?*

Each study is reported along with its results and a discussion. The paper concludes with an outlook on future work (see “Conclusion and outlook” section).

Altogether, this paper provides novel insights into the design of gaze-based, MW-aware audio guides for the scenario of panorama viewing. These insights include findings on the adaptation and re-validation of the MW classifier and MW annotation method proposed earlier^14^ for real world scenarios with a long focus distance. The results show that our classifier reaches high performance even without eye vergence features.

## Related work

### Mind wandering

Mind wandering (MW) can be defined as “a shift in the contents of thought away from an ongoing task and/or from events in the external environment to self-generated thoughts and feelings”^15^. This article investigates the detection of MW during audio-guided panorama viewing, i.e., on a combined audio-visual stimulus. Based on studies which suggest that states of boredom may be conducive to MW^16,17^, we evoke MW by intentionally playing long, non-engaging audio content.

Theories and studies on MW consider it as not merely a passive state but one that involves complex cognitive processes. One key aspect is the interplay between the default mode network (DMN)—a brain network implicated in self-referential thinking^18^—the executive control system, and the salience network^19^. When salient events or stimuli are detected, the salience network suppresses the activity of the DMN and redirects attentional resources toward the relevant stimulus. In our study, we bring the user back to the task by imposing a salient effect on the audio stimulus.

MW has been associated with lower recall^8^, negative impact on learning outcomes^20^, and poor comprehension during memory and reading tasks^21^, respectively. MW also has an influence on an individual’s affective state, leading to unhappiness in many cases^22^, but can be followed by positive affective states if thoughts are future- and self-related^23^. In general, although MW can be positive in some situations (e.g., for creativity^24^), it can be challenging for interactive systems because it can lead to missed information, delayed responses, and an overall decrease in the quality of interactions. Therefore, it is critical for an intelligent system to be aware of its user’s shifts in attention.

Previous work has attempted to detect shifts in users’ attention in different scenarios, including driving^25^ and predicting the viewing times of exhibits in museums^26^. Results have shown that users may either overtly or covertly shift their attention away from a primary task (e.g., viewing a panorama)^27^. An overt shift in attention occurs when a person intentionally attends to the source of information by moving their eyes to fixate on the source^27^. In contrast, a covert shift in attention is not always followed by corresponding changes in eye movement. Different modalities have been used for detecting MW, including electroencephalogram (EEG) signals^28^, skin conductance/temperature^29^, eye gaze^13,30^ and acoustic prosodic information^31^. Despite these efforts, detecting MW can be challenging because it requires inferring about an internal state with only a few overt markers^32^.

### Pervasive eye tracking

Pervasive eye tracking^33^ can provide insights into human behavior that complements other methods (e.g., interviews or external observation^34^). The eyes can be tracked pervasively using different devices such as head-mounted^35^ or remote^36^ eye trackers and webcams^37^. Recent advances in pervasive outdoor eye tracking have enabled gaze-based interactions with everyday objects in outdoor spaces^38^. For example, researchers have used mobile eye trackers for creating dynamic audio narratives based on gaze while tourists explored a city panorama^39^. While well-designed audio narratives may lower the risk of a user starting to mind wander, they can neither prevent MW completely nor bring the user back to the task.

### Gaze-based detection of mind wandering/attention shift

Previous work has demonstrated the possibilities of using eye gaze for detecting shifts in attention. For example, thermal imaging and both stimulus-dependent (e.g., gaze transitions) and -independent gaze features (e.g., number of fixations) have been successfully combined for attention classification^10^. Content-dependent and -independent gaze features have also been used to detect MW during computerized reading^40^ with fewer and longer fixations found to be critical gaze signatures of MW in reading^41^. Beyond reading research, content-independent gaze features have been found to be useful for MW detection for narrative film comprehension^42^, tutoring systems^30^ and lecture viewing^32^. Goldberg and colleagues^43^ found that using gaze, head posture, and facial expression features, extracted with a machine vision-based approach, provided good estimations of the manual ratings of visible indicators of students’ (dis)engagement in learning. In addition, Krasich and colleagues investigated gaze allocation during MW when participants studied images of urban scenes^44^ and found that probe-caught MW was associated with fewer and longer fixations, greater fixation dispersion, and more frequent blinks. Unlike previous work^44^, which only focused on the effect that resulted from the visual stimulus, we focus on MW when individuals are exposed to a combination of visual and auditory information.

Recent work from Bixler and D’Mello^45^ trained and compared within-domain models and cross-domain models with five different domains (i.e., illustrated text, narrative film, video lecture, naturalistic scene, and reading text) based on a dataset that uses the probe-caught method, which actively stops the participants during the task and collects their response. The models were trained with gaze data within a 40-s time window prior to the thought probes. The results indicated that cross-domain model training could obtain accuracies (AUROCs of 0.56 to 0.68) comparable to the within-domain models (AUROCs of 0.57 to 0.72). Although cross-domain models seem promising, the models cannot be applied directly to our scenario as we focus on detecting MW within a 1-s time window.

Huang and colleagues^14^ proposed the real-time detection of attention shift from the visual task towards internal thoughts during video viewing using eye vergence features within a 1-s time window. Their best detection model with only vergence features (trained with a Random Forest classifier) achieved an average F1 of 0.74 which was a 12.1% improvement over the state-of-the-art^30,32,40,41^. Notably, eye vergence features, which measure the rotation of both eyes, have some limitations. Compared to the focus distance between the screen and the user in video viewing, the degree of changes in eye rotation (from focus to “staring to space”) will be less if objects are located further away. Since many real world scenarios (e.g., a tourist fixating on the top of a mountain that is located far away) have a longer focus distance, it is yet unclear whether vergence features can be used for classifying MW. As such, we consider both commonly used gaze features (e.g., fixation durations, saccade velocities) and eye vergence features for MW detection. To the best of our knowledge, there is no prior research on real-time gaze-based MW detection during the interaction with a city panorama.

### Mind wandering annotation

Since MW is an internal cognitive state, it can be challenging to reliably annotate instances in an eye movement data set as “MW” or “not MW”. Previous research has employed probe-caught^32,40^, self-caught^8^ and fine-grained annotation methods^14^ to label episodes of MW. In a probe-caught method, the system (or experimenter) actively stops the participants during the task to inquire about their MW experience. Here, it can be difficult to annotate the start and end of MW even if participants are aware of their own MW. The self-caught method requires participants to consciously report their own MW experience and may provide a more precise annotation with respect to the end of an episode of MW. However, this method is still limited because the start of the MW is not taken into account, and participants may also fail to notice when MW occurs^8^.

In contrast, the fine-grained annotation method^14^ is based on the assumption that participants who are on-task will be quicker to notice a change in the stimulus. Here, participants are exposed to a stimulus and asked to press a key once they notice a change (i.e., blurring), after which the stimulus returns to its normal state (i.e., deblurring). While the self-reporting methods have a start time that has to be chosen arbitrarily, this method has the advantage that it can be assumed that an MW event had already started at the start of the blurring effect. This method allows researchers to record the time interval between the start (i.e., when the stimulus begins to blur) and the end (i.e., when participants react) of an MW event. We adopt the fine-grained annotation method in our experimental design.

## Pre-study: parameters for data annotation

Different to previous work^14^ that used blurring of a visual stimulus (i.e., a video) to annotate MW, in this paper, we randomly blur the auditory stimulus (voice) of an audio guide. We conducted a pre-study in order to determine the type of audio blurring and the parameters that can be used for the annotation. Specifically, we manipulated the fade-out mode and fade-out speed of the audio. Three levels were considered for fade-out mode: changing volume only (decrease from 100 to 0.01%), changing speech speed only (decrease from 180 to 135 words per minute (wpm)), and changing the volume and speech speed at the same time. For each fade-out-mode, there were three levels (fast, medium and slow) of fade-out speed, which translates to a per second decrease of 9/4.5/3 wpm for changes in speech speed and a decrease of 5/2.5/1.67% per second for changes in volume. The study was approved by the University’s institutional review board (IRB, the ETH Zurich Ethics Commission, approval number: EK 2019-N-96) and has been performed in accordance with the Declaration of Helsinki.

### Methodology

#### Participants

An a priori power analysis was conducted using G*Power version 3.1.9.7^46^ to determine the minimum sample size required to test the study hypothesis. Results indicated that the required sample size to achieve 80% power for detecting a large effect, at a significance criterion of $\alpha =0.05$, was $N = 18$ for a 3 × 3 mixed factorial ANOVA analysis. Eighteen participants (7 females, mean age of 29.11 years, ranged 19–37 years) with normal hearing ability were recruited for the pre-study. All participants were asked to sign a written informed consent form prior to starting the experiment. Participants did not receive any monetary compensation and were allowed to abort the experiment at any time they wanted.

#### Materials

Participants viewed a city panorama displayed on a 28-inch computer screen while receiving audio instructions through headphones. A total of 18 audio clips, each lasting for approximately 20 s, were used. In order to control for the pronunciation, emotion, consistency of the speech-speed fade-out effect, and delivery across all stimuli, the audio clips were generated with the IBM Watson Text-to-Speech (TTS) service^47^ (in American English). We used a voice generated by deep neural networks in order to obtain a more human-sounding synthesized speech.

#### Procedure

Participants were instructed to listen to audio descriptions of different features of a city panorama image of Geneva, Switzerland. An audio fade-out effect started at random time intervals (between 2 and 5 s) during the audio playback. Note that a short time interval was chosen on purpose in order to avoid MW, since we study reaction time only in the pre-study. The participants’ task was to press the spacebar key on the keyboard immediately after noticing a change in the audio. At this stage, the audio went back to normal until the next blurring effect started. The audio volume or speech speed was reduced dynamically until the user noticed the fade-out effect. All the participants were able to recognize the blurring effect in all trials and reacted accordingly. Participants randomly experienced all nine combinations of fade-out mode and fade-out speed twice, resulting in a total of 18 measures per participant. The entire experiment lasted for 15 min and also included a series of debriefing questions about the users’ experience.

#### Results

**Fig. 1:**
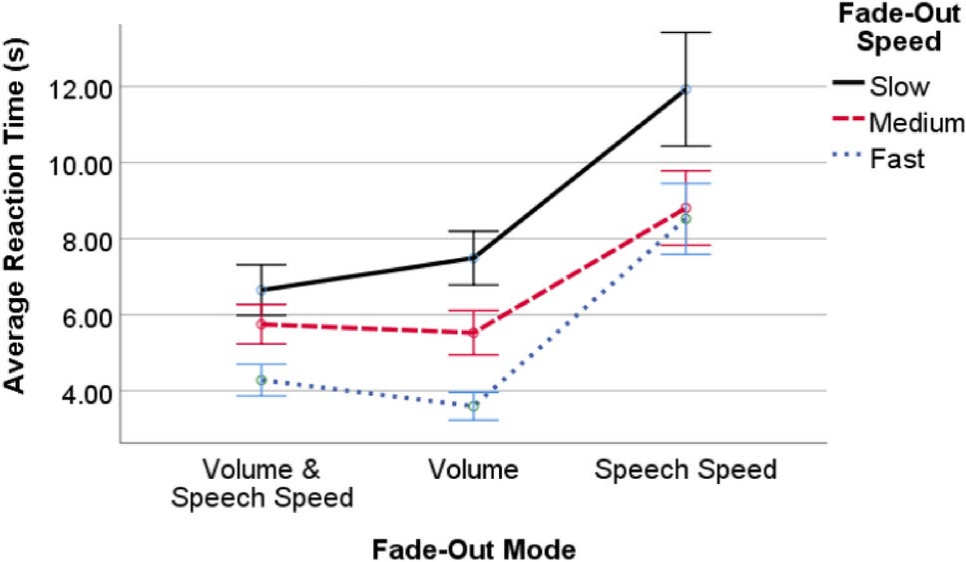
Results of the pre-study: Average reaction times for different combinations of fade-out mode and fade-out speed. Error bars indicate standard error.

A 3 (fade-out mode) × 3 (fade-out speed) mixed factorial ANOVA with Greenhouse–Geisser correction revealed a significant main effect for fade-out mode ($F_{1.293,21.979} = 32.669,$ $p <0.001$) and fade-out speed ($F_{1.424,24.210} = 18.941,$ $p <0.001$ ). The interaction between fade-out mode and fade-out speed was not significant ($p = 0.199$). Overall, participants were quicker to react to volume fade-out with a fast fade-out speed. Results show that, on average, participants reacted within 3.59 s ($SD = 1.56$ s) to the fast fade-out speed, 5.52 s ($SD = 2.47$ s) to the medium fade-out speed, and 7.49 s ($SD = 3.00$ s) to the slow fade-out speed. Here, pairwise comparisons with Bonferroni correction further revealed significant differences in reaction times between fast and medium $(p < 0.001, d = 0.93),$ fast and slow $(p < 0.001, d = 1.63),$ and medium and slow $( p =0.002, d = 0.71)$ when fading-out by volume only (see Fig. 1). Notably, 44% of participants also reported hearing artifacts for the fade-out modes that included a change in speech speed.

We selected the volume fade-out mode combined with fast fade-out speed to be used in the detection of MW in study 1. This choice was based on the fact that participants reported hearing artefacts for change of speech speed. Fast fade-out speed was chosen because it had the lowest SD. Here, a lower SD implies less variation and a lower chance of false positives (FP) or false negatives (FN) in the collected data. The average reaction time of this combination (3.59 s) is used as a parameter in the data analysis of Study 1.

## Study 1: detecting mind wandering

The goal of the first study was to collect a reliable ground truth for training a classifier capable of distinguishing between two states (fully focused or MW) from short 1-s windows of eye movement data that can be implemented in an interactive system. With this, we address the research question *(RQ1) Can eye vergence features be used for classifying MW in scenarios with longer focus distances?*

### Methodology

#### Participants

A total of 27 participants (16 females, M = 22.81, range 18–33) participated in the study. All participants were recruited via the DeSciL participant recruitment platform of ETH Zurich and were required to have normal or corrected to normal vision (with contact lenses) and normal hearing ability. All participants were asked to sign a written informed consent form prior to starting the experiment. Participants were paid 30 CHF for 75 min of participation and could abort the experiment at any time they wanted. The study was approved by the University’s IRB (approval number: EK 2019-N-96) and has been performed in accordance with the Declaration of Helsinki.

#### Materials

**Fig. 2:**
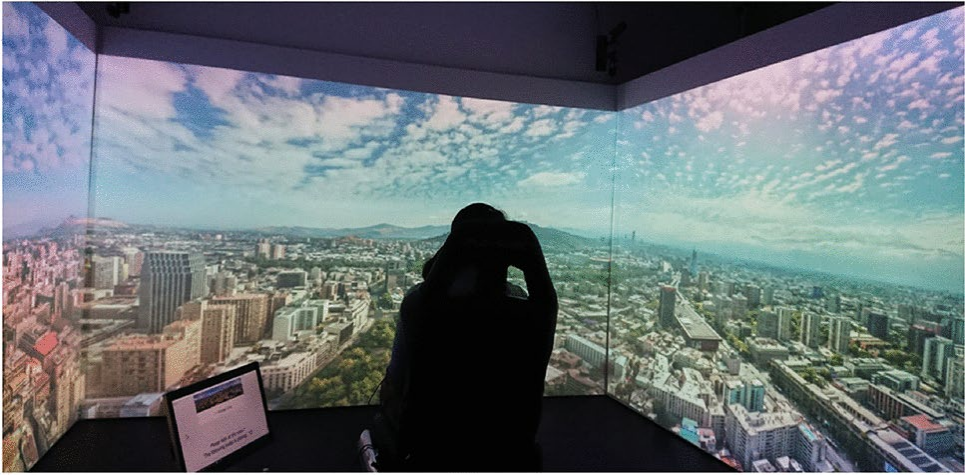
Experiment setup in the CAVE (study 1). The participant is wearing a head-mounted eye tracker and headphones. A keyboard is in front of the participant and connected to the experimenter’s computer.

The experiment was conducted in a CAVE (Cave Automatic Virtual Environment) with three large projection walls (see Fig. 2). A seat was located at the center of the room (1.8 m away from the front-facing wall). The lights were turned off, and participants wore the 120 Hz SMI Eye Tracking Glasses (binocular eye tracking with a reported accuracy of 0.5°) and headphones during the experiment. Gaze data in the coordinate system of the projection wall were obtained in real-time by matching the front-facing video of the eye tracker to a reference image using an ORBm feature detector^38,48^.

One panorama for each of six different cities (i.e., Barcelona, Santiago, Paris, Melbourne, Chicago and Buyeo) was used in order to vary the landscape and building types across trials. Within each panorama, we have defined five Areas of Interest (AOIs). The AOIs were selected from the salient features within the panoramic view. For each panorama, we prepared four short audio clips lasting between 29 and 32 s and one long audio clip lasting between 9 min 46 s and 10 min 5 s. We used the short audio clips to collect data while participants were fully focused on the task. The long audio clips were used to collect data on MW. The content of the audio descriptions included touristic information obtained from the official web pages of the selected cities or buildings (if available) and Wikipedia^49^. All audio clips, generated using TTS^47^, were making explicit reference to the AOIs selected within each panorama.^1^

After the experiment, participants were asked via a questionnaire about their familiarity with the city (on a 7-point Likert scale) and the way they would like to be alerted in case of MW.

#### Procedure

Upon arriving at the laboratory, participants read an information sheet about the experiment and signed a consent form. They were then seated on the chair and equipped with the eye tracker, headphones, and a keyboard. Participants were instructed to place the keyboard at a location permitting them to press the space bar without looking at it. At this stage, the experimenter calibrated the eye tracker and told participants that they were allowed to move their head during the experiment to explore the views on their left and right-hand sides.

We first collected data to be labelled as fully focused. Participants were shown two panoramas with short audio clips and were instructed to pay close attention to the panoramas and the audio descriptions. Second, we collected data on MW by showing participants the four remaining panoramas accompanied by long audio descriptions. Here, participants were instructed to press the spacebar key when they detected that the audio started to fade out. Based on the pre-study results (see “Pre-study: parameters for data annotation” section), the fade-out mode volume with fast fade-out speed was chosen. The fade-out frequency was chosen randomly between 15 and 30 s. This choice was motivated by related work^14^ who chose from an interval between 10 and 20 s for a reaction time that was found to be around 2 s shorter than ours in their pre-study. All panoramas were shown in a pseudo-random order across participants, and the eye tracker was re-calibrated before the viewing of the third and fifth panoramas. At the end of the experiment, participants were asked to complete the questionnaires.

#### Dataset labeling

The collected data were segmented into 1-s windows without overlaps. Focused data were collected from the first part of the study, where the participants were listening to short audio clips and instructed to pay close attention to the panoramas and the audio description. A total of 2644 s labelled as “fully focused” were generated.

**Fig. 3:**
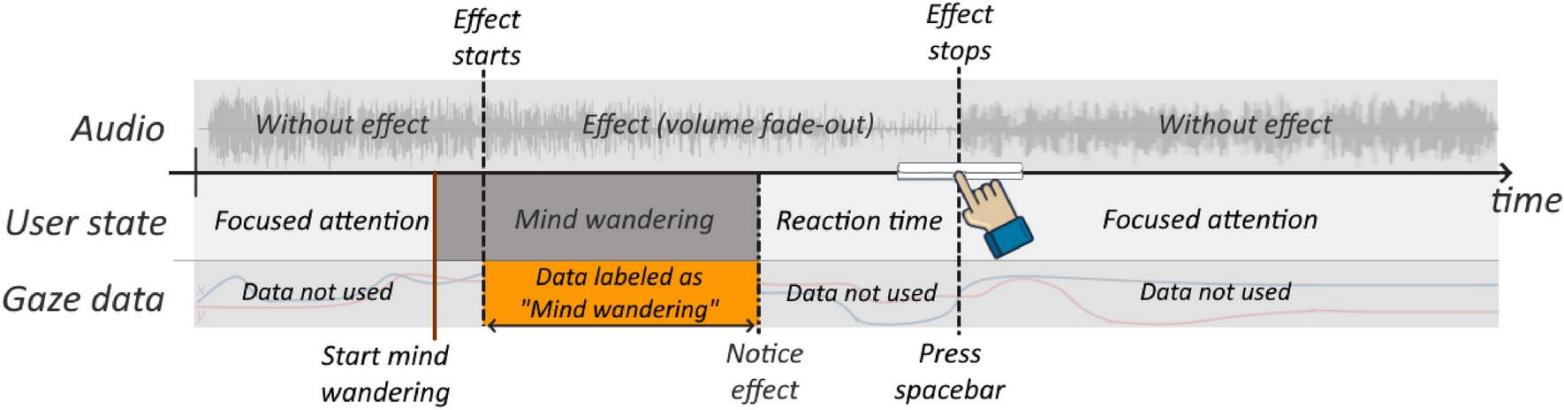
MW annotation (study 1). Participants listened to an audio which started to fade-out at random intervals. The starting time of the fade-out effect is indicated by the line “Effect starts”. Participants were instructed to press the spacebar once they noticed the effect to terminate the volume fade-out effect (“Effect stops”). The assumed reaction time of 3.59 s was determined in the pre-study (“Pre-study: parameters for data annotation” section). Gaze data between “Effect starts” and “Notice effect” were annotated as MW .

MW data were obtained from the second part of the study, where long audio descriptions were played, and participants had to press the spacebar key when they detected that the audio started to fade out. Figure 3 illustrates the annotation principle for MW instances. The line “Effect starts” indicates the time the audio started to fade out. The line “Effect stops” indicates the time participants pressed the spacebar key, which made the audio return to normal. The “Effect starts” line annotated an approximate start time of the participants’ MW state. However, participants could have started MW anytime before that. This is illustrated by the fact that the “Start mind wandering” line is drawn before the “Effect starts” line. The “Effect stops/Press spacebar” and the “Reaction time” give us an approximate end time for the MW state. Since participants may not have been in a MW state while reacting to the fade-out effect (“Reaction time”), we did not use the gaze data during this period.

**Fig. 4:**
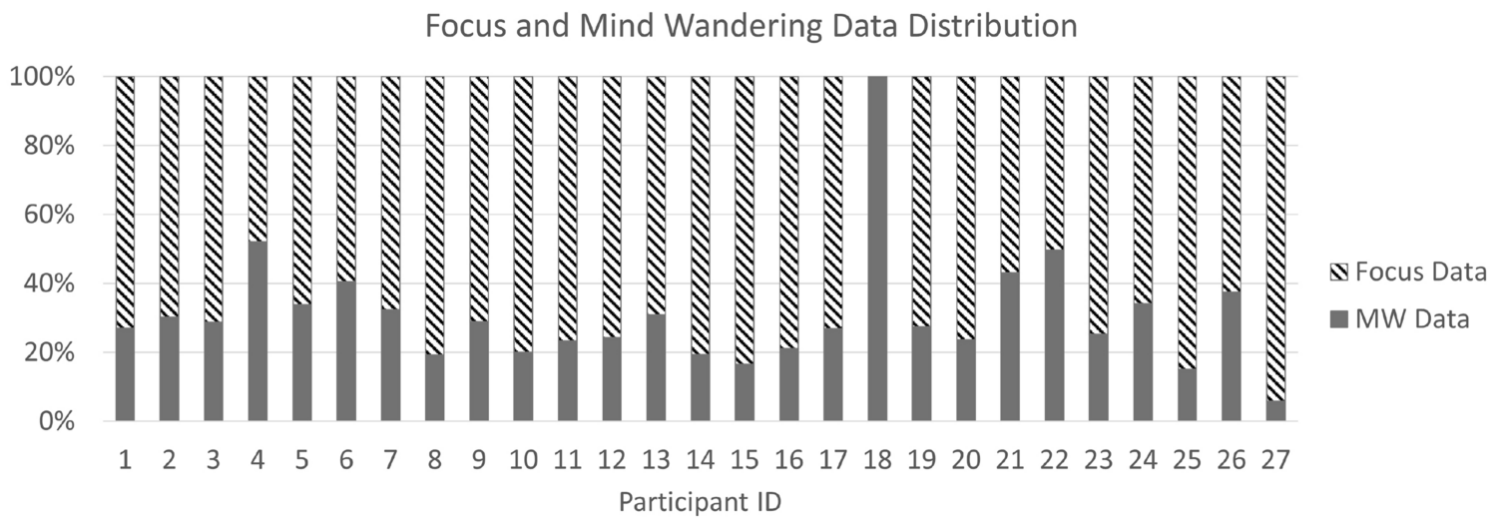
Distribution of focused and MW data for each participant.

We considered a duration of 3.59 s (the mean reaction time from the pre-study) as the expected reaction time for participants between noticing an effect and pressing the space bar. We then labelled as MW all data between the start of the effect until when the participants noticed the change. A total of 1123 s were labelled as MW. On average, participants had 29.4 MW instances ($$SD = 14.6),$$ which is on average 31.0% ($$SD = 16.9\%)$$ of the total number of instances observed for each participant (see Fig. 4). Note that participant 18 has 0% focused data collected due to an error with the eye tracker calibration. We were able to retain his/her MW data as we re-calibrated the eye-tracker before the third and fifth trials started. Compared to other more conservative thresholds with the mean reaction time, we found that more conservative thresholds would have produced a highly imbalanced dataset for classification. Using the mean resulted in 2644 s of fully focused and 1123 s of MW data out of approximately 20 h of collected data. In contrast, using a 95% confidence interval would have resulted in 443 s of MW, and a threshold of 3 SDs would have resulted in only 193 s of MW.

#### Feature extraction and classifier training

Fixations were detected with the I-DT algorithm^50^ using dispersion of 1$^{\circ }$ and min duration 100 ms as thresholds. Saccades were computed from the fixations. In total, 37 features were extracted from the gaze data (see Table 1). This gaze feature set is adapted from previous work and covers fixation, saccade^14,32,41^ and vergence^14^ features.

**Table 1:** List of features used for training the classifier. Numbers in brackets indicate the total number of features for a certain type of eye movement.

Type of eye movement	Attribute
Fixation (11)	Duration (ms)*
	Number of fixations per second
	Percentage of fixation duration
	Duration ratio of fixation over saccade
	Radii of minimal bounding circles
Saccade (22)	Duration (ms)*
	Length (px)*
	Velocity (degrees per second)*
	Number of saccades per second
Vergence (4)	Disparity of gaze pair (Mean, SD) (px)
	Centroid distance of gaze point sets (Mean, SD) (px)

Lines marked with an asterisk (*) indicate a set of 7 features based on mean, min, max, median, standard deviation (SD), skewness, and kurtosis of the respective attribute.

We excluded some of the gaze features suggested by related work^14,32,41^. These features were excluded because they cannot reliably be detected in our scenario. For example, fixation dispersion was not considered because the dispersion threshold of I-DT was set to the accuracy of the eye tracker (as reported in the specification, i.e., 0.5° of visual angle) and this resulted in noisy data. Furthermore, features related to blinks were not considered because it is hard to distinguish if the missing gaze data are caused by blinking, failure in mapping the gaze to the reference image (e.g., looking at the sky leads to not enough good features used for tracking) or the SMI eye tracker lost track of a participant’s gaze. We did not perform within-participant feature normalization since our goal is to build a user-independent model that can be generalized to unseen users.

We trained four machine learning models for MW detection: Random Forest, Support Vector Machine (SVM), XGBoost, and Multilayer Perception (MLP). The choice of models was based on successful reports of the performance of these models in the relevant literature (see “Gaze-based detection of mind wandering/attention shift” section). The hyperparameters of the four machine learning models were tuned with the following setup: we carried out five iterations of 3-fold cross-validation on the full dataset. To avoid overfitting, we selected the hyperparameter based on the number of iterations during which it was consistently chosen as the optimal parameter across 15 trials. In each fold, nine randomly selected participants were left out so that the data from 18 participants were used for training and the remaining data for testing. We used a leave-9-participants-out cross-validation because some of the participants had an unbalanced class distribution. Finally, we used the following hyperparameters for the classifiers: Random Forest with 100 trees, SVM with a polynomial kernel and a regularization parameter (C) of 20.0, XGBoost with a learning rate of 0.1 and a maximum depth of 10, and MLP with two hidden layers, a learning rate of 0.5 (with decay), and a momentum of 0.3.

We then tested the classifier with another round of five iterations of 3-fold cross-validation to obtain the average performance of the classifier. The random seeds used in this testing phase differed from those used in the hyperparameter tuning phase, which could also help in avoiding the problem of hyperparameter overfitting.

#### Analysis of questionnaires

SPSS was utilized for all statistical analyses of the questionnaire data. The codes and themes of the open-ended questions’ responses were created by one of the authors and iterated to revise, combine or expand. The name for each theme was carefully considered to ensure the names truly reflect the meanings. The frequencies of the themes were calculated and reported.

### Results

#### Classifying mind wandering

In total, we trained eight classification models, which are the four machine learning methods trained with data that either included or excluded eye vergence features. For each classification model, the 15 individual performance results (five iterations, 3-fold cross-validation) were averaged. Table 2 presents the results of these classifiers, including the Area Under the Curve (AUC), the weighted F1 score, and the false positive rates (FP), as well as the precision and recall of MW detection. All models had an AUC larger than 0.7 and FP rate below 0.08. The Random Forest classifiers had an AUC of 0.8 and an F1 Score of 0.8, which outperformed the SVM, XGBoost, and MLP classifiers across different feature combinations. Therefore, we selected and applied the Random Forest classifier with vergence in Study 2. Results of an info gain attribute evaluation with Weka^51^ further revealed that the top five predictive features were the number of fixations per second, mean fixation duration, maximum fixation duration, duration ratio of fixation over saccade, and percentage of fixation duration.

**Table 2:** Summary of the performance of the mind wandering classification from gaze features with and without vergence.

	Vergence	Evaluation Metrics				
		AUC	F1 Score	FP MW	Precision MW	Recall MW
Random Forest	With	0.800 (0.025)	0.806 (0.027)	0.040 (0.008)	0.845 (0270)	0.507 (0.058)
	Without	0.803 (0.025)	0.806 (0.023)	0.047 (0.011)	0.829 (0.037)	0.516 (0.052)
SVM	With	0.708 (0.023)	0.788 (0.028)	0.033 (0.012)	0.854 (0.048)	0.450 (0.048)
	Without	0.708 (0.023)	0.787 (0.028)	0.034 (0.012)	0.853 (0.048)	0.450 (0.049)
XGBoost	With	0.741 (0.025)	0.808 (0.024)	0.058 (0.010)	0.803 (0.028)	0.540 (0.056)
	Without	0.728 (0.026)	0.794 (0.024)	0.076 (0.014)	0.754 (0.039)	0.532 (0.055)
MLP	With	0.796 (0.030)	0.803 (0.027)	0.040 (0.013)	0.845 (0.041)	0.500 (0.061)
	Without	0.798 (0.030)	0.805 (0.028)	0.039 (0.010)	0.850 (0.029)	0.503 (0.064)

Ablating these critical features led to the anticipated decline in model performance. Specifically, for the Random Forest model trained with vergence features, the AUC decreased from 0.708 to 0.630, while the F1 score fell from 0.788 to 0.720. The false positive rate remained relatively stable at 0.034, but precision decreased from 0.854 to 0.793, and recall dropped from 0.450 to 0.295.

Based on the weights derived from the 15 individual SVM models trained with vergence features, we compiled a list of features consistently ranked among the top 10 weighted features. These features align closely with those identified through the information gain attribute evaluation. Notably, they include metrics such as maximum, mean, median, standard deviation, and percentage of fixation duration, as well as the number of saccades per second and the duration ratio of fixation to saccade.

#### Familiarity with the cities

Participants were least familiar with Santiago (*M* = 1.41, *SD* = 0.73) and Buyeo (*M* = 1.37, *SD* = 1.34), followed by Melbourne (*M* = 2.56, *SD* = 2.03), Chicago (*M* = 3.48, *SD* = 2.01), Barcelona (*M* = 5.30, *SD* = 1.54) and Paris (*M* = 5.41, *SD* = 1.69). These familiarity results were used as a criterion for the selection of panoramas in Study 2.

#### Preferred interaction

Participants also reported the type of interaction they would prefer if this had been an interactive system capable of detecting MW while they viewed the panorama (one or more suggestions could be listed). Table 3 presents a summary of the responses. Most participants (9 out of 27) suggested either playing an audio cue (e.g., beeping sound, loud noise, alert sound or warning audio such as “wake up”), switching to another topic (9), or changing the volume (8). Only two participants preferred not to have the system respond in such situations.

**Table 3:** Suggestions for system responses when MW is detected (questionnaire after study 1).

	Number of respondents (out of 27)
**Question**: “Imagine you are mind wandering while using the audio guide system, and the system knows that you are mind wandering. What kind of responses should be provided by the system? (you can list more than 1 suggestion)”	
Audio cues	9
Switch topic/Ask whether to continue	9
Volume change	8
Visual cue	8
No response needed	2
Haptical feedback	1
Clear separation of segments	1
Changing the voice of the narrator	1
Pause until focused	1
Stop the audio guide	1

### Discussion

#### Performance of the classifiers

With the Random Forest classifier, we were able to predict MW with an AUC of 0.80. Comparing the performance of classifiers trained with and without vergence features helps in answering the research question “*(RQ1) Can eye vergence features be used for classifying MW in scenarios with longer focus distances?*”. Contrary to previous work^14^, we found that different features of fixations and saccades, and not vergence features, were the best predictors of MW. This difference may be related to the fact that our participants viewed static panoramas in a CAVE environment rather than videos^14^. Here, it is possible that focus distances in the CAVE (1.8 m) and video (~ 60 cm) led to substantial differences in vergence and the eventual prediction of MW. Notably, our classifiers were somewhat conservative in predicting instances of MW. This was evident from the recall rate, which showed that approximately 50% of detected MW was correctly classified. Interestingly, this was offset by the low FP rates, which showed that only 5% of detected MW instances were false alarms. A low FP rate is important because it can facilitate the interaction (e.g., reduce annoyance) with users leading to higher UX.

#### Suggested system responses for an interactive system

The purpose of our question regarding the type of responses of a potential MW-aware system was to inform the design of the interaction. Among the suggestions we received, audio cues and switching topics/asking whether to continue were most frequently mentioned by the participants and therefore could potentially be included in future studies. Previous work has also suggested that adaptive systems should avoid “patronization”^52^, therefore, one potential solution can be granting the user control over the interaction by asking participants whether to switch topics instead of switching topics automatically.

Visual cues and volume changes were suggested by the same number of respondents (eight participants) as interaction methods for notifying the user. Depending on the use case scenarios, adding visual cues on the visual stimulus might not be ideal if the goal is to develop a system that also works outside a CAVE without the need to place a display in between the environment and the user^39^. These interaction ideas can be tested in future studies to find out how to assist users in effectively regaining focus.

## Study 2: re-validation of the MW classifier in CAVE

The goal of study 2 was to further validate the MW classifier trained from gaze data with eye vergence when it is applied to the scenario of detecting MW in real time. Because we did not want to interrupt the participants with probe messages during data collection in Study 1, the training data did not contain any kind of self-reported labels. Therefore, instead of using the labelling method used in Study 1, Study 2 validates the model with unseen data that are annotated based on quiz questions. Different from the method of self-reporting the MW experience, for which it can be difficult to determine the start or even the end of MW, the quiz method helps in determining these because the starting and ending times of the “critical sentence” are known. The critical sentence is the only sentence of the audio that contains the answer to the quiz question. Thus, by assuming that the quiz questions will be answered correctly only if the user is not MW when the critical sentence is mentioned, the correlation between correct/false answers and the amount of time having mind-wandered can be used to further assess the validity of the MW classifier.

### Methodology

#### Participants

A total of 32 participants (16 females, M = 25.30, range 20–39, 1 discarded) with normal or corrected to normal vision (with contact lenses) and normal hearing ability participated in the study. A minimum sample size of N = 31 is required to achieve 90% power for detecting a large effect size, at a significance criterion of $\alpha =0.05$. Thus, the obtained sample size is adequate to test the study hypothesis. Participants from Study 1 could not participate in Study 2. Participants were recruited via the same platform as in Study 1. All participants were asked to sign a written informed consent form prior to starting the experiment. The participants were paid 25 CHF per 60 min and told that they were allowed to abort the experiment at any time. The study was approved by the University’s IRB and has been performed in accordance with the Declaration of Helsinki.

#### Materials

The study was carried out in the same CAVE with a similar hardware setup as in study 1. In this study, an additional monitor was used to display the quiz questions and it was carefully placed at a location that did not block the panorama view when not in use. Moreover, participants interacted with the small monitor with a mouse instead of a keyboard. The MW Random Forest classifier trained from gaze data with eye vergence (see “Study 1: detecting mind wandering” section) was running in the background and performed second-based classification in real time. The detection results were saved into files for further analysis.

We selected 3 (Santiago, Melbourne, Chicago) out of the 6 panoramas used in study 1 based on the (low) level of familiarity of participants in Study 1, including only the cities which also had a minimum of five tourist attractions in view. For each panorama, we selected five different buildings as target tourist attractions and prepared a short audio clip for each building (between 104 and 126 s), as well as a multiple-choice quiz question. The quiz questions, offering four choices based on the contents of the short audio clips, were designed in a way that they shared a similar difficulty and could not be answered without listening to the audio. The quiz questions were always related to a critical sentence within the audio. To eliminate the influences caused by differences in memory skill, the last sentence of the audio clip was always selected as the critical sentence, and the most important information (e.g., the construction year of a site that is being asked) never appeared in the last few words of the sentence. Apart from the 4 choices, we also provided an option of “I am not sure” to avoid random guesses.^2^

To measure the perceived difficulty of each quiz question, participants were asked: “Compared to the question asked in the warm-up task, how difficult is the quiz question?” with an 11-point Likert scale ranging from − 5 to + 5 (with negative ratings representing lower difficulty and 0 representing the same difficulty).

#### Study design and analysis

All participants completed the three trials (one for each city panorama) in a pseudo-random order (Latin squares randomization). The experiment required approximately 45 min to complete. Statistical analyses were performed with R^53^.

#### Procedure

Participants were given an information sheet and asked to sign the consent form. Next, participants sat on the chair at the center of the CAVE and were asked to put on the headphones and eye tracker and place the mouse next to themselves. The experimenter then calibrated the eye tracker (3-point calibration) and told participants that they were allowed to move their head during the experiment to explore the views on their left and right-hand sides. The experiment started with a warm-up task that contained an introduction audio, a warm-up quiz question, and a question about perceived difficulty. On each trial, participants were asked to press a start button once they were ready to begin. Each trial consisted of five audio clips. The questions (quiz and perceived difficulty) for each audio were asked directly after the audio. Each trial took approximately 12 min (including the time needed for answering the quiz).

#### Measurements

We investigated whether the quiz questions were answered correctly or incorrectly. Specifically, we encoded the choice of the correct answer as “Correct” and the choice of all the other answers (including the option “I am not sure”) as “Incorrect”. Only 50.9% (208 out of 409) of the questions were answered correctly.

We measured the percentage of time mind-wandered within the critical sentence and used it for the analysis. This is equivalent to the number of seconds being classified as MW divided by the total duration (in seconds) that is used for classification within the critical sentence.

### Results

#### Missing data

Data from a number of trials (14.79%) were excluded due to hardware disconnection, and data from one of the participants (male) had to be entirely discarded. As a result, we collected a total number of 409 valid instances contributed by 31 participants. On average, the number of valid instances collected from each participant was 13.19 (ranging from 6 to 15). Fortunately, the valid instances were also distributed evenly among the three city panoramas, with the number of valid instances ranging between 133 and 140.

#### Perceived difficulty

**Fig. 5:**
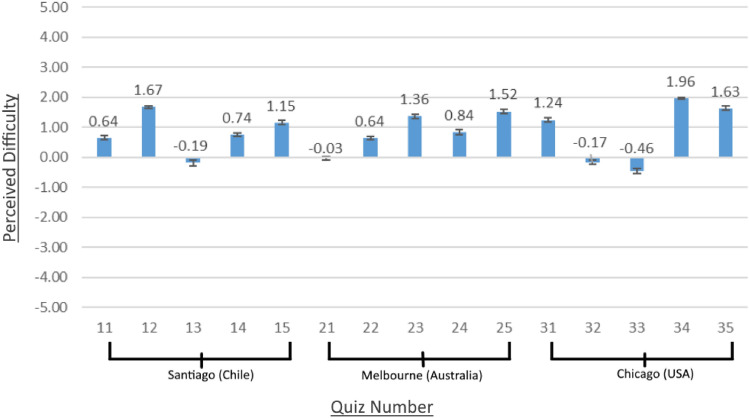
Perceived difficulty of the quiz questions compared to the warm-up question in study 2 (± standard error) (− 5 = less difficult; 0 = same; 5 = more difficult). The x-axis indicates the quiz number: tens for Santiago, twenties for Melbourne, and thirties for Chicago. There are five quizzes per city, each of which contains one question. Sequence of trials (cities) was pseudo-randomized, quizzes within a trial occurred in a fixed sequence.

Figure 5 shows the average perceived difficulty of different quiz questions with the mean values ranging from − 0.46 to 1.96. Overall, participants reported a relatively low difference in perceived difficulty between the quiz questions and the warm-up question. For most of the quiz questions, the perceived difficulty is slightly higher (i.e., more difficult) than the warm-up question, which was answered correctly by all participants. The results indicate that the quiz questions are similar in terms of perceived difficulty, and therefore data from all the quiz questions was used for the analysis.

#### Percentage of time being classified as MW

**Fig. 6:**
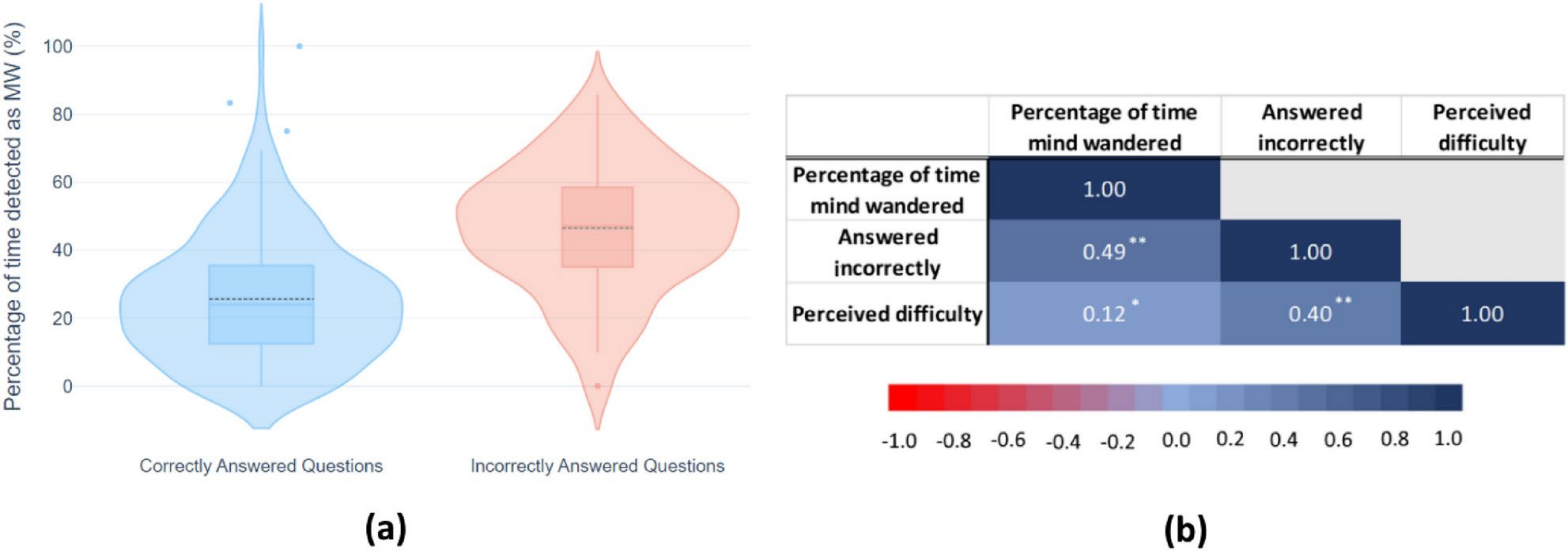
(**a**) Overview of the data distribution of the percentage of time detected as MW for the correctly and incorrectly answered questions. The dotted lines within the box plot represent the mean values. (**b**) Results of study 2 that show the matrix of repeated measure correlation (*rmcorr*) between the three variables, including the percentage of time that is detected as mind wandered, whether the quiz question was answered correctly or incorrectly and the perceived difficulty of the quiz question. $$p<0.001**,\; p <0.05*$$.

We visualized the descriptive statistic of the percentage of time classified as MW based on whether the quiz questions were answered correctly or not. Figure 6a shows the probability density of the data (smoothed by a kernel density estimator) together with box plots showing summary statistics. On average, participants have 25.65% $(SD = 18.48\%)$ of time being classified as MW when they answered the quiz correctly while having 46.51% $(SD = 18.76\%)$ of time classified as MW when they answered the questions incorrectly.

#### Repeated measure correlation

Applying simple correlation to non-independent observations or aggregated data (e.g., data that are averaged within participants) can produce biased results due to a violation of the independence assumption and/or differing patterns between participants versus within-participants. Bakdash and Marusich^54^ proposed the repeated measure correlation (*rmcorr*) to tackle this limitation. The *rmcorr* takes non-independence into account without averaging or aggregating the observations. Since our observations violated the independence assumption used in simple correlation, we analyze the data with the *rmcorr* R package provided in^54^.

Results of *rmcorr* (see Fig. 6b) indicate that there is a positive relationship between the percentage of time mind wandered and incorrect answering ($r_{rm}$(377) = 0.49, 95% CI [0.41, 0.56], $p < 0.001$), and between perceived difficulty and incorrect answering ($r_{rm}$(377) = 0.40, 95% CI [0.31, 0.48], $p < 0.001$). We also found a weaker positive relationship between the percentage of time mind wandered and perceived difficulty ($r_{rm}$(377) = 0.12, 95% CI [0.02, 0.22], $p < 0.05$).

Note that we also ran point-biserial correlations by averaging the observations within participants and obtained similar results as *rmcorr*. Using this method, we found a positive relationship between the average percentage of time mind wandered and the percentage of questions answered incorrectly ($r_{(29)}$ = 0.61, 95% CI [0.32, 0.79], $p < 0.001$) and between average perceived difficulty and the percentage of questions answered incorrectly ($r_{(29)}$ = 0.60, 95% CI [0.31, 0.79], $p < 0.001$). However, the relationship between the average percentage of time mind wandered and average perceived difficulty is not significant ($r_{(29)}$ = 0.30, 95% CI [− 0.07, 0.59], $p > 0.05$).

### Discussion

Study 2 aimed at providing a validation of the MW classifier used as the back-end system of the audio guide, based on participants’ ability to answer quiz questions about a critical sentence played in a touristic audio guide. This study helps to address the research question “*(RQ2) Can MW be detected reliably for uninterrupted audio-guided panorama viewing?*”.

Our results suggest a positive answer to RQ2: we observed a positive correlation between the percentage of time MW and incorrect answering. This implies that the more time is detected as MW, the more likely the participants answered the question incorrectly. With our assumption that the question will be answered correctly only if the user is not MW when the critical sentence is played, the result supports our MW detection model’s ability to detect MW correctly.

## Conclusion and outlook

This work contributes to providing gaze-based interaction that proactively helps in regaining and maintaining the attention of the user for the use case of audio-guided panorama viewing. To achieve this goal, we investigated how to enable systems to detect MW from eye movements in a use case with long focus distance. With the presented novel MW annotation method for combined audio-visual stimuli, we trained and validated an MW classifier with the annotated MW data, re-validated the classifiers in the CAVE with unseen users, and collected a list of potential interaction methods for providing feedback to the users.

We have demonstrated the possibility of detecting MW in a 1-s time window from eye movements during audio-guided panorama viewing (study 1). We believe we are the first to detect MW for this kind of interaction scenario. Due to the difference in focus distance between the scenarios used by Huang et al.^14^ and this work, we focused on comparing the models trained with and without eye vergence features. One particularly interesting finding is that vergence-based eye movement features did not contribute notably to the classifier performance (different to previous work^14^). Future work, especially for scenarios containing visual objects located at different focus distances, can investigate whether including the focus distance as a feature in the machine learning model helps in improving detection accuracy. In addition, the current fine-grained annotation method is mainly based on the average reaction time of different users. Obtaining individual reaction time might help in reducing the amount of noise within the dataset, and potentially improve the classifier performance.

Since the classifiers trained with and without the eye vergence features perform similarly, one could choose whether to include them by considering the pros and cons of the classifier. On the one hand, the eye vergence features were proven to be helpful with a short focusing distance^14^. On the other hand, including them did not harm the classifier with a long focusing distance. This implies that use cases with a focusing distance smaller than the one tested here will potentially benefit from the use of eye vergence features. However, including and computing the eye vergence features requires extra computational power, which might be a limiting factor for real-time applications. If the target device has limited computational resources, it might be beneficial not to include the eye vergence features.

Results of study 2 (n = 31) re-validated the MW classifier in the panorama viewing scenario. Overall, the model cross-validation carried out in Study 1, and the analysis of the MW classifier performance in Study 2 based on the quiz method provides sufficient evidence that the model trained in this work is valid.

Previous research by Kwok et al.^39^ demonstrated the feasibility of applying mobile eye tracking research results that were obtained in a controlled lab environment to the real world environment. Results show that users were capable of using the system in the real world without loss in system usability. On the other hand, the authors pointed out that sunlight affects the reflective properties of the infrared light used by the eye tracker. This leads to an increased amount of missing gaze data in the outdoor environment. In their outdoor study, there were around 11.8% of gaze data missing. This missing data can lead to not being able to classify some of the MW moments. Despite this, our real-time second-based classification methodology stands resilient, offering the advantage of detecting within a given second. Furthermore, even though our results were obtained in the CAVE, the low contribution of vergence features to the classification result makes us optimistic that it may be possible to train similar classifiers for the real world. However, this needs to be confirmed in future studies.

Our work takes a step in the direction of constructing intelligent MW-aware audio guides that are capable of detecting users’ level of attention and helping them regain focus. With the list of interaction ideas we collected in Study 1, future studies can be carried out to answer research questions such as what are adequate ways to interrupt the interaction and how to make use of the information that were obtained by the MW classifiers.

## Data Availability

Data privacy regulations prohibit the deposition of individual-level data to public repositories, and ethical approval does not cover the public sharing of data for unknown purposes. The minimal data set consists of the aggregated data required to replicate the studies’ statistical findings reported. It is available from the corresponding authors upon reasonable request.

## Footnotes

1 Example: “Flinders Street Station, which is the railway station located in front of you, is one of Melbourne’s most recognized landmarks located on the corner of Flinders and Swanston Street in the central business district.”

2 Example question for Melbourne: “What kind of art installation is installed at the base of Eureka Tower?” Options: “real beehive, beehive statues, bee statues, white box with bees, I am not sure”

## Code availability

Code for analysis is provided as part of the replication package. It is available at https://zenodo.org/records/14128251.
